# Supplementary material for: Development of the awareness, skills, knowledge: General (ASK-G) scale for measuring cultural competence in the general population
Source: PLoS One. 2022 Sep 15;17(9):e0274505. doi: 10.1371/journal.pone.0274505 (PMC9477359; doi:10.1371/journal.pone.0274505)
Supplement: S1 Appendix — (DOCX) [file pone.0274505.s001.docx]

**Appendix A. Factor Loadings of Original 81-Item ASK-G Scale.**

| Item | Varimax Rotated Factor Loadings | | | | Promax Pattern Matrix Loadings | | | |
| --- | --- | --- | --- | --- | --- | --- | --- | --- |
|  | 1 | 2 | 3 | 4 | 1 | 2 | 3 | 4 |
| I am a cultural being. | .478 | .176 | .250 | .406 | .474 | -.037 | .113 | .339 |
| My cultural heritage has shaped who I am. | .194 | .136 | .058 | **.766** | .152 | .009 | -.097 | **.777** |
| My beliefs and values are rooted in my cultural background. | .199 | .262 | .143 | **.648** | .097 | .172 | -.010 | **.623** |
| I have a clear ethnic label that I use to identify myself. | .316 | .108 | .233 | .421 | .296 | -.061 | .133 | .380 |
| When I use an ethnic label to describe myself, I know what that label means to me. | **.633** | .210 | -.057 | .248 | **.728** | .004 | -.254 | .192 |
| My culture has an impact on the way I see the world. | .127 | .106 | .193 | **.730** | .051 | -.016 | .081 | **.731** |
| My culture has an impact on the way I think of others. | .010 | .064 | .275 | **.635** | -.094 | -.026 | .217 | **.634** |
| My culture affects the way I behave toward others. | .054 | .079 | .459 | **.502** | -.076 | -.033 | .435 | .464 |
| White Americans have a culture. | .094 | .233 | -.126 | .398 | .040 | .237 | -.261 | .402 |
| All people have some cultural biases. | .198 | .327 | -.030 | .225 | .125 | .323 | -.165 | .184 |
| My culture has shaped the way I see the world. | .223 | .146 | .163 | **.632** | .169 | .011 | .034 | **.617** |
| I sometimes lack awareness about cultural context in my interactions with people from backgrounds other than my own. | -.033 | .444 | .369 | .146 | -.291 | **.520** | .321 | .053 |
| My cultural perspective is one of many. | .399 | .444 | .215 | .186 | .288 | .367 | .062 | .080 |
| There is no one “right” cultural perspective. | .178 | **.552** | .026 | .190 | .005 | **.617** | -.138 | .112 |
| There is no one “normal” culture. | .271 | **.562** | .066 | .077 | .115 | **.600** | -.095 | -.023 |
| Racism affects everybody, not just underrepresented ethnic groups. | .222 | **.574** | -.161 | .074 | .096 | **.664** | -.349 | .005 |
| My cultural group membership has affected the opportunities that have been available to me. | .141 | .187 | **.580** | .348 | -.025 | .072 | **.558** | .262 |
| My cultural values shape my assumptions about what is normal and abnormal. | .049 | .115 | .258 | **.612** | -.060 | .029 | .183 | **.602** |
| My life experiences (e.g., upbringing, education, socioeconomic status) impact my worldviews. | .306 | .292 | .069 | .390 | .248 | .205 | -.085 | .344 |
| My culture gives me a unique perspective of the world. | .421 | .041 | .220 | .392 | .459 | -.183 | .117 | .351 |
| Culture plays a role in the opportunities (e.g., education, occupation, healthcare, housing) that people have. | .205 | .388 | .354 | .272 | .030 | .344 | .256 | .178 |
| I have said things that offended someone else’s culture without realizing it until someone pointed it out. | -.055 | .173 | .478 | .142 | -.240 | .166 | **.505** | .073 |
| I notice when I am the only person of my culture in a room or at a gathering. | .173 | .157 | .187 | .363 | .108 | .071 | .104 | .329 |
| In the past, I have made disparaging remarks about cultural groups other than my own that I now realize were problematic. | -.086 | .150 | .453 | .192 | -.267 | .147 | .480 | .136 |
| When I think of new foods (e.g., cow tongue or deep fried butter), I understand that my disgust is partly due to my cultural background. | -.015 | .484 | .260 | .270 | -.267 | **.566** | .169 | .192 |
| When people from cultural backgrounds different from mine tell stories, it is difficult for me to listen without grounding their stories in my own experience. | -.042 | .176 | .410 | .225 | -.214 | .165 | .413 | .169 |
| When I make a cultural misstep, I see that moment as a learning opportunity. | .256 | **.596** | .305 | .025 | .038 | **.623** | .182 | -.112 |
| There is room for me to grow in cultural competence. | .358 | **.610** | .155 | .033 | .192 | **.621** | -.012 | -.095 |
| I will make cultural missteps in the future. | .019 | .419 | .223 | .280 | -.192 | .473 | .131 | .214 |
| I know about specific behaviors or routines that are specific to cultural groups other than my own (e.g., differences in how people greet each other). | **.646** | .226 | .328 | .151 | **.665** | -.022 | .196 | .037 |
| I know some history about people that belong to cultural groups different from my own. | **.615** | .305 | .086 | .136 | **.645** | .126 | -.091 | .044 |
| I know some history about my own cultural group. | .477 | .367 | .052 | .211 | .449 | .256 | -.127 | .130 |
| I know the difference between prejudice and discrimination. | **.541** | .207 | -.039 | .191 | **.611** | .039 | -.208 | .136 |
| Anyone is capable of discriminating against others. | .320 | .487 | -.236 | .020 | .271 | **.530** | -.429 | -.040 |
| Individuals have multiple identities (e.g., religious, gender), some of which give them privilege and some of which place them at risk for marginalization. | .332 | .435 | .190 | .155 | .213 | .388 | .051 | .057 |
| The services received by underrepresented ethnic groups in the United States systematically differ from those of middle- or upper-class White Americans. | .263 | .416 | .258 | .202 | .117 | .377 | .139 | .107 |
| Institutionalized racism exists. | .185 | .485 | .207 | .248 | .000 | .498 | .074 | .159 |
| Underrepresented ethnic groups in the United States have access to fewer opportunities than middle- or upper-class White Americans. | .206 | .417 | .484 | .063 | .003 | .386 | .427 | -.066 |
| White Americans in the United States have access to more opportunities than people that belong to underrepresented ethnic groups. | .102 | .361 | .428 | .231 | -.103 | .343 | .371 | .137 |
| I am familiar with religious beliefs and practices of cultural groups other than my own. | **.628** | .158 | .216 | .171 | **.688** | -.089 | .081 | .083 |
| I have learned about the history of a cultural group other than my own. | **.667** | .270 | .270 | .051 | **.690** | .045 | .130 | -.070 |
| Health disparities based on culture exist in the United States. | .224 | .446 | .416 | .068 | .029 | .425 | .339 | -.058 |
| I am familiar with important customs of a cultural group other than my own. | **.623** | .177 | .280 | .154 | **.662** | -.069 | .154 | .055 |
| Family is defined differently by different cultures. | .293 | .486 | .121 | .137 | .159 | .480 | -.029 | .042 |
| I can name five inspirational people that come from cultural backgrounds different than mine. | .418 | .339 | .322 | .002 | .339 | .230 | .226 | -.117 |
| I can name five inspirational people that come from my cultural background. | .296 | .218 | .244 | .195 | .237 | .111 | .158 | .125 |
| Colonialism and other oppressive forces are still at work across the world. | .257 | .377 | .173 | .258 | .139 | .334 | .043 | .182 |
| Some people have dietary restrictions specific to their cultural or religious upbringings. | .448 | **.512** | -.144 | .109 | .402 | .491 | -.362 | .030 |
| I can understand slang terms used by people from a variety of cultures. | .388 | .158 | .341 | .177 | .358 | -.013 | .267 | .094 |
| It is okay for people to adopt characteristics from cultural groups other than their own (e.g., White Americans wearing dread locks, a Latinx individual wearing a bindi). | .196 | .370 | .083 | .059 | .090 | .382 | -.021 | -.013 |
| It is okay for people to dress up as Native Americans for Halloween. | -.033 | -.045 | -.041 | .221 | -.026 | -.063 | -.061 | .250 |
| It is okay for people that live in different countries (e.g., a return missionary that served in Colombia) to claim that ethnic identity (e.g., “I’m part Colombian now”). | .085 | .068 | .362 | .012 | .008 | .005 | .385 | -.049 |
| It is okay to wear clothes from a culture different from your own if you are doing so respectfully (i.e., wearing a properly wrapped sari to an Indian friend’s wedding). | .326 | .449 | .019 | .106 | .237 | .436 | -.141 | .025 |
| Cultural competence is a lifelong journey rather than something with an end goal. | .334 | **.515** | .135 | .234 | .193 | .489 | -.039 | .137 |
| I can comfortably adjust my personal space to the needs of people from a variety of cultural backgrounds. | .449 | .311 | .382 | -.054 | .379 | .180 | .303 | -.184 |
| When I say something that is offensive to another person, I can apologize even if I do not fully understand how I have offended them. | .272 | **.647** | .160 | .051 | .066 | **.700** | -.001 | -.074 |
| I can recognize the problem with applying stereotypes to specific cultural groups. | **.509** | .388 | .133 | .064 | .472 | .276 | -.024 | -.042 |
| I am able to take the perspective of a person from a culture other than my own. | **.582** | .399 | .154 | .070 | **.556** | .258 | -.016 | -.045 |
| I am able to adapt my body language when communicating with someone from a culture other than my own. | .487 | .198 | .272 | .145 | .483 | .013 | .167 | .054 |
| I am able to adjust my communication style when communicating with someone from a culture other than my own. | **.558** | .411 | .068 | .009 | **.541** | .300 | -.105 | -.100 |
| I can adapt the words I use so they are culturally appropriate for people from a variety of culture backgrounds. | .423 | .426 | .197 | .002 | .338 | .356 | .066 | -.114 |
| I have attended ceremonies/celebrations (e.g., holiday celebrations, weddings, funerals, birthdays) from cultures different than my own. | **.569** | .250 | .349 | .017 | **.558** | .051 | .247 | -.106 |
| I have taken the time to learn about ways of being that are different from my own (e.g., religious traditions, coming-of-age ceremonies, medicinal approaches). | **.696** | .183 | .222 | .177 | **.764** | -.085 | .071 | .080 |
| I read books, historical or fictional, about cultural groups/traditions different from my own. | .360 | .181 | .471 | .104 | .289 | .019 | .426 | -.002 |
| I listen to lectures or podcasts about cultural topics. | .228 | .005 | **.594** | .100 | .163 | -.172 | **.627** | .013 |
| I change my communication style depending on the cultural background of the person/people I’m interacting with. | .191 | .085 | .492 | .225 | .101 | -.054 | .484 | .152 |
| I modify my behavior to align with the cultural values of the people I interact with. | .184 | .402 | .425 | .105 | -.010 | .378 | .361 | -.010 |
| I have joined a group that advocates for the rights of people in cultural groups different from my own. | .155 | .003 | **.740** | .057 | .043 | -.160 | **.814** | -.045 |
| I openly speak a language other than my native language. | .178 | -.137 | **.654** | .141 | .139 | -.348 | **.728** | .072 |
| When I hear a racist joke, I confront it. | .419 | .232 | .468 | -.045 | .352 | .078 | .422 | -.172 |
| I regularly attend social action events (e.g., protests, town hall meetings) in my community. | .088 | -.105 | **.766** | .138 | -.010 | -.285 | **.866** | .058 |
| I engage in advocacy work that advances the wellbeing of marginalized populations (e.g., homeless people, low income children). | .241 | .057 | **.644** | .109 | .148 | -.117 | **.670** | .009 |
| I confront racist comments made by family members. | .325 | .347 | .458 | -.095 | .194 | .269 | .410 | -.232 |
| I confront racist comments made by friends. | .355 | .308 | .456 | -.098 | .247 | .208 | .411 | -.232 |
| I confront racist comments in public settings made by strangers. | .354 | .172 | **.533** | -.091 | .280 | .023 | **.526** | -.216 |
| I can conceal disgust when trying a new food (e.g., pig’s eyes, gizzards) that has been presented to me as a delicacy in order to be respectful toward the person presenting it to me. | .208 | .129 | .444 | .142 | .119 | .011 | .428 | .062 |
| I refrain from using certain words and phrases that I know may be offensive. | .381 | **.504** | .166 | -.025 | .262 | .480 | .025 | -.146 |
| If I use an offensive term, I catch myself in the moment and correct my language. | .227 | .456 | .264 | .102 | .058 | .453 | .157 | -.003 |
| I am able to track the amount of time I spend talking within a group of people in an effort not to dominate conversations. | .197 | .309 | .356 | .335 | .047 | .238 | .267 | .256 |
| When I notice I have been dominating a group conversation, I am able to step back so others’ voices can be heard. | .454 | .368 | .126 | .087 | .409 | .270 | -.022 | -.009 |
| I make a point to amplify the voices of marginalized peoples by repeating their comments and giving them credit for their ideas. | .395 | .307 | .447 | .029 | .294 | .179 | .378 | -.098 |
| When I make a racist remark, I take time to reflect on the intention behind my comment and try to think of other ways I might get my point across. | .088 | **.538** | .334 | .066 | -.163 | **.607** | .251 | -.050 |
| When I witness others make a cultural misstep, I respond with understanding. | .336 | .388 | .362 | .080 | .204 | .311 | .267 | -.039 |
| I am confident when meeting people from cultural groups that I have little familiarity with or knowledge of. | **.513** | .305 | .229 | .155 | .484 | .145 | .090 | .054 |

*Note*. Bolded values indicate the values above .50 in each of the different solutions. When comparing Varimax and Promax solutions to one another, only 9 items total differed; 5 items were contained in the Varimax solution that were not contained in the Promax solution and 4 items were contained in the Promax solution that were not contained in the Varimax solution.
