# Supplementary material for: Development of the awareness, skills, knowledge: General (ASK-G) scale for measuring cultural competence in the general population
Source: PLoS One. 2022 Sep 15;17(9):e0274505. doi: 10.1371/journal.pone.0274505 (PMC9477359; doi:10.1371/journal.pone.0274505)
Supplement: Attachment — Submitted filename: PONE-D-22-02341 Response to Reviewers.docx [file pone.0274505.s002.docx]

PONE-D-22-02341 – Development of the awareness, skills, knowledge: general (ASK-G) scale for measuring cultural competence in the general population

| Reviewer Comment | Authors’ Response |
| --- | --- |
| Reviewer #1: I appreciate the opportunity to review this manuscript. This research addresses the measurement of cultural competence in the general population, a relevant and little explored topic. However, some aspects require clarification. | We thank the reviewer for their helpful feedback! We have worked hard to integrate your suggestions throughout the manuscript, which has improved the overall quality of the manuscript and its potential impact. We hope that these changes address any remaining concerns you may have. |
| 1) In the line 58, the authors argue "no know measures are designed for a general population". However, it is necessary to consider antecedents on measurements of intercultural personality and multicultural knowledge. The definition of these constructs and the items present in related measurements tools are similar to proposed in the manuscript reviewed. These aspects should be included in the manuscript.  2)In line 128, the authors mention a tool to measure CC in the general population. They should delve into it. | We agree with the reviewer that the introduction could benefit from more detail regarding limitations of prior measures of cultural competency. We have included additional information regarding prior measures, their factors, and limitations in the second to last paragraph of the “Cultural Competency” section. We have maintained the prior flow of the introduction as we believe that it is important to first inform the reader of issues regarding the conceptualizing, definition, and measurement of cultural competency before describing established measures.  We also provide further clarification to the statement in the first paragraph of the introduction (starting on line 58) to strength the rational for the current study.  “While cultural competency measures for the general population exist, several limitations hinder their utility in assessing training and research outcomes (i.e., narrow focus on specific components, poor predictive validity, and generalizability of factor structure).” |
| 3) The section about the panel comments should be shortened as it is not a qualitative analysis of the information reported but only a selection of vignettes. | We thank the reviewer for this suggestion. We choose to retain this portion of the methods as the expert reviewers helped not only inform the contents of the survey but also helped reduce the length of the questionnaire so that analyses would be feasible. We provide additional information regarding this choice in the subsequent response. We felt that this provided needed transparency regarding the contents of the survey. |
| 4) The argument for using Exploratory Factor Analysis (EFA) was the wide variety of conceptualization and definition of cultural competence. However, the items were developed using the Sue and Sue model, which has a precise definition of CC. Also, in line 155, they indicate, "we expect to find three factors". This hypothesis justify starting with a confirmatory model, and then (if it is necesary) an exploratory approach. | Thank you for this comment and allowing us the opportunity to further elaborate on the methodological approach. Originally, we did evaluate CFA analyses for each of the subscales as well as the expected full 3-factor solution using Mplus, which resulted in poor model fit even with the least constrained model (a congeneric measurement model), which is why we turned to parallel analysis to determine the number of factors. We have now included these results in the manuscript and apologize for not including them earlier. |
| 5) The authors estimated a sample to carry out EFA with 36 items, but the original questionnaire has more than 80. This aspect requires clarification since the sample size should consider the total number of items used in the first EFA. A sample of 200 people for an 80-item questionnaire might be insufficient. | Conducting a power estimate for an EFA is not only difficult, but guidelines vary according to experts from “2 subjects per variable” (Kline, 1994), “5 subjects per variable or 100 subjects, whichever is larger” (Hatcher, 1994), “300 subjects, though fewer works if correlations are high among variables’ Tabachnik & Fidell, 2001), “20 subjects per factor” (Arrindel & van der Ende, 1985). Further, characteristics within the data (such as factorability, uniformly high communalities, cross-loadings, missingness, etc.) all impact the sample size needed to estimate the number of factors. We could have conceptualized sample size according to number of subjects per variable, the total number of subjects, or the number of subjects per factor. According to these various guidelines, we could have concluded that sample sizes of 160, 400, 300, and 60 could have been acceptable. We are aware that a sample size of 200 is possibly insufficient for the 80-item analysis, which is why we only retained a much smaller subset of items in the final factor structure. There was no reason for us to retain all of the items developed, instead, we only needed to retain the best items.  If there are clearer guidelines for sample size estimation with EFA that we are unaware of, please do let us know and we will incorporate that into a further revision of the manuscript. |
| 6) The authors performed an EFA considering an orthogonal rotation, which assumes that the factors are independent of each other. This decision is not theoretically justified. They report moderate and high correlations between the factors, and the content of items does not suggest independent factors. An oblique rotation might be evaluated and the theoretical meaning of the items within the factors need to be revisited (there are very similar items in different factors, for example, "I able to adjust my communication style when communicating with someone from another culture…" (Knowledge) and "I refrain certain words and phrases that I know may be offensive" (Others Awareness)) | We used varimax rotation because it often results in factor structures with relatively few large loadings for each factor (Kaiser, 1958), and allows interpretation of subscales as independent factors (rather than as a set of intercorrelated factors). This makes the factor structure more easily interpretable when constructing mean scores, and allows assessment of factor subscales separately. However, the reviewer does have a point. We did think that the subscales should be correlated and in fact found that they were in the final step, since all of the items measure some aspect of cultural competency. For this reason, we now present results from both the varimax and promax (an oblique rotation) factor solutions in Appendix A. We still, however, retain the results from the varimax solution due to the relative similarity between the two solutions. |
| 7) The authors must include the first EFA with all items applied. This information could be in a supplementary file. | All items and the results from the first EFA are now included in Appendix A. |
| 8) Given the nature of the items, some of them could load in two or more factors in a significant way (for example, loads >0.4). Report all factor loads. | We now report all factor loadings for items that were retained (updated Table 4), and similarly include all factor loadings even for items not retained in Appendix A. |
| 9) The analysis section is integrated with the results section. I suggest adding an analysis section in the methodology for greater clarity. | We have added a Data Analysis section in the methods to separate analysis from results. We have moved sentences from the results into this section as appropriate. |
| 10) I suggest incorporating some general estimation of the level of cultural competence (general and for each factor) achieved by the participants. | We have already reported the mean cultural competence scores for each factor as well the general factor for the entire population in Table 5. We now mention this in a sentence on p. 22. |
| 11) There is no theoretical definition of the factors in the final factor solution. | We thank the reviewer for their feedback. We have added an analytic plan section that better articulates that the theorized factor loadings were first examined with a CFA that fit the data poorly. Subsequent analyses arrived at the final factor solution through data driven exploratory analyses. This clarification has also been added the final paragraph of the introduction. |
| 12) The discussion should place the findings in context by comparing them with other relevant literature. This aspect requires further development. | We agree with the reviewer that the discussion needed to be more closely tied to prior theoretical conceptualizations of cultural competency and established measures. We have made changes to better frame our findings within the context of the existing literature. |
